# The acidic amino acid-rich C-terminal domain of VanabinX enhances reductase activity, attaining 1.3- to 1.7-fold vanadium reduction

**DOI:** 10.1016/j.bbrep.2022.101349

**Published:** 2022-09-16

**Authors:** Tri Kustono Adi, Manabu Fujie, Nori Satoh, Tatsuya Ueki

**Affiliations:** a Laboratory of Molecular and Cellular Physiology, Graduate School of Integrated Sciences for Life, Hiroshima University, 1-3-1 Kagamiyama, Higashi-Hiroshima, Hiroshima, 739-8526, Japan; b Chemistry Department, Faculty of Mathematics and Natural Sciences, State Islamic University of Malang, Malang, 65145, Indonesia; c DNA Sequencing Section, Okinawa Institute of Science and Technology Graduate University, Onna, Okinawa, 904-0495, Japan; d Marine Genomics Unit, Okinawa Institute of Science and Technology Graduate University, Onna, Okinawa, 904-0495, Japan

**Keywords:** Next-generation sequencing, Metal accumulation, Metal transporters, Metal binding, Enzyme kinetics

## Abstract

Ascidians accumulate extremely high levels of vanadium (V) in their blood cells. Several V-related proteins, including V-binding proteins (vanabins), have been isolated from V-accumulating ascidians. In this study, to obtain a deeper understanding of vanabins, we performed *de novo* transcriptome analysis of blood cells from a V-rich ascidian, *Ascidia sydneiensis samea*, and constructed a database containing 8532 predicted proteins. We found a novel vanabin with a unique acidic amino acid–rich C-terminal domain, designated VanabinX, in the database and studied it in detail. Reverse-transcription polymerase chain reaction analysis revealed that VanabinX was detected in all adult tissues examined, and was most prominent in blood cells and muscle tissue. We prepared recombinant proteins and performed immobilized metal ion affinity chromatography and a NADPH-coupled V(V)-reductase assay. VanabinX bound to metal ions, with increasing affinity for Cu(II) > Zn(II) > Co(II), but not to V(IV). VanabinX reduced V(V) to V(IV) at a rate of 0.170 μM per micoromolar protein within 30 min. The C-terminal acidic domain enhanced the reduction of V(V) by Vanabin2 to 1.3-fold and of VanabinX itself to 1.7-fold in *trans* mode. In summary, we constructed a protein database containing 8532 predicted proteins expressed in blood cells; among them, we discovered a novel vanabin, VanabinX, which enhances V reduction by vanabins.

## Highlights

• A novel vanadium-binding protein was identified from a vanadium-rich ascidian.
• This protein named VanabinX does not bind strongly to V(IV).
• VanabinX can reduce V(V) to V(IV) in a NADPH/GR/GSH cascade.
• The acidic C-terminal domain of vanabinX enhances V(V)-reduction of vanabins in trans mode.

## 1 Introduction

Ascidians, also known as tunicates or sea squirts (Chordata, Urochordata, Ascidiacea), accumulate extremely high concentrations of vanadium (V) [[1], [2], [3]]. Species belonging to the family Ascididae accumulate a maximum of 350 mM V, corresponding to approximately 10^7^ times the concentration of V ions in natural seawater [4,5]. V ions in the +5 oxidation state in natural seawater are taken up by ascidians, reduced to the +3 oxidation state, and stored in the vacuoles of vanadocytes, which are blood (coelomic) cells [[6], [7], [8]].

Many genes involved in V accumulation and reduction have been identified in ascidians using biochemical and molecular biological methods, mainly using the V-rich ascidian species *Ascidia sydneiensis samea*, which can accumulate 12.8 mM V in its blood cells. V-binding proteins called vanabins were first identified in blood cells by ion exchange chromatography [9,10]. Three additional vanabins and another V-binding protein were later purified through metal ion affinity chromatography and expressed sequence tag (EST) analysis [[11], [12], [13]]. Enzymes in the pentose phosphate pathway, which produce two NADPH molecules per cycle, were identified using specific monoclonal antibodies and polymerase chain reaction (PCR) analysis [14,15], and the NADPH produced was revealed to be involved in V redox [16]. Membrane transporters for V, sulfate, chloride, and protons were also identified through degenerate PCR [[17], [18], [19]]. The localization and function of these proteins suggested that a V accumulation and reduction pathway existed in blood cells [20,21]; however, this pathway remains poorly understood, and the related genes and proteins are unknown.

Vanabins comprise a unique gene family that has been identified only from V-rich ascidians. After the discovery of Vanabin1 and Vanabin2 in *Ascidia sydneiensis samea* [9,10], efforts were made to identify more vanabins in this species and in other organisms. We searched the draft genome database of the ascidian *Ciona intestinalis* type A (*C. robusta*) [22] and found five vanabins in this species [23]. We first applied biochemical approaches to identify the novel vanabin VanabinP in *A*. *sydneiensis samea* [11], and then successfully identified the novel vanabins Vanabin3 and Vanabin4 through EST analysis of *A*. *sydneiensis samea* blood cells [12,24], as well as AgVanabin1 and AgVanabin2 through EST analysis of *Ascidia gemmata* intestinal tissue [25]. Vanabins were later found to act as V(V)-reductases [16,26,27]. Vanabins have 18 conserved cysteine residues, and form nine disulfide bonds to create a bow-shaped structure [28], although some of these disulfide bonds are reversible in redox [16]. In our model, V(V) ions were readily reduced to V(IV) in the cytoplasm by vanabins, and V(IV) ions were stabilized by vanabins [20]. Thus, vanabins act as both V reductases and V chaperones.

Information about the proteins involved in the V accumulation and reduction pathways remains limited. Under the assumption that next-generation sequencing technology would allow us to determine huge numbers of sequence tags at lower cost within a shorter time, in the present study we performed a transcriptome analysis of *A*. *sydneiensis samea* blood cells and a BLAST search against a public protein database to identify V-related proteins and examine their metal-binding and metal-reducing abilities.

## 2 Materials and methods

### 2.1 RNA extraction from ascidians

Adults of the ascidian *Ascidia sydneiensis samea* were collected at Kojima Port, Okayama, Japan (34°26′36.3″ N 133°48′43.5″ E). They were cultivated in an aquarium with flowing natural seawater at the Marine Biological Laboratory, Graduate School of Integrated Sciences for Life, Hiroshima University, Hiroshima, Japan (34°21′55.3″ N 133°12′55.9″ E) and fed regularly with the diatom *Chaetoceros* sp. Blood was extracted from each individual and diluted with Ca^2+^- and Mg^2+^-free artificial seawater (460 mM NaCl, 9 mM KCl, 32 mM Na_2_SO_4_, 6 mM NaHCO_3_, 5 mM HEPES, and 5 mM EDTA, pH 7.0). Blood cells were collected by centrifugation at 300×*g* for 10 min at 4 °C. Giant cells were removed by sucrose density gradient centrifugation, as this type of cell contains highly acidic materials that adversely affect RNA extraction [4]. Blood samples from four ascidian individuals were separately treated and used in this study (Supplementary Table 1). Tissues other than blood cells were manually excised from each body part using scissors and tweezers.

RNA was extracted from blood cells by a cesium ultracentrifugation method [29]. Blood cells from each individual were homogenized in a solution containing 4 M guanidine thiocyanate, 0.1% sodium *N*-lauryl sarcosinate, 5 mM EDTA, and 40 mM Tris-HCl at pH 7.0 (GTC solution) and further homogenized with an ultrasonicator. The homogenate was added to a solution of 50% cesium trifluoroacetate and 100 mM EDTA and centrifuged (100,000×*g*, 16 h, 15 °C) by an ultracentrifuge (Optima TLX; Beckman). Precipitated RNA was recovered and dissolved in sterilized RNase-free water.

RNAs from the other tissues were extracted using the acid guanidine phenol–chloroform method [30]. Tissues were homogenized in GTC solution using a hand homogenizer. Acid phenol solution was prepared by mixing 0.15 M sodium acetate (pH 4), 50% citrate-saturated phenol, and 10% chloroform/isoamyl alcohol (24:1 stock). The homogenate was mixed with an equal volume of acid phenol solution, shaken vigorously for 15 s, and incubated on ice for 15 min. The mixture was centrifuged at 15,000×*g* for 30 min. The supernatant was recovered, and RNAs were precipitated by isopropanol. Precipitated RNAs were dissolved in sterilized RNase-free water.

### 2.2 Construction of the cDNA library, DNA sequencing, and protein prediction

RNAs were quantified by the Agilent Bioanalyzer. One hundred nanograms of RNA were used to make a cDNA library with the NEB Next Ultra Directional RNA Library Prep Kit for Illumina. Each cDNA Library was divided in half. One set of cDNA was normalized following a method described by Matvienko et al. [31]. The other set was sequenced without normalization. Multiplex analysis using a MiSeq DNA sequencer (Illumina, Inc., CA, USA) provided paired-end reads (300 bp × 2). Raw sequence reads are deposited in the DDBJ public database under accession number DRA008341.

Shotgun sequence data from each of ascidian individual were assembled with the Trinity software ver. 20140413 [32]. The abundance of each transcript was estimated using the RSEM toolkit and calculated as aligned read counts per transcript [33]. Coding sequences were predicted using TransDecoder with the option of minimum length of 65 amino acids. The details of sequencing and assembly are summarized in Supplementary Table 2.

To facilitate the homology search, a customized protein database was constructed using a subset of NCBI protein databases. This database includes nonredundant protein sequences from two ascidian genera (*Ciona* and *Ascidia*) and a larvacean (*Oikopleura*), nematode (*Caenorhabditis elegans*), zebrafish (*Danio rerio*), sea urchin (*Strongylocentrotus*), budding yeast (*Saccharomyces*), and bacterium (*E*. *coli*) downloaded on 24 July 2014. The total number of protein sequences was 151,632.

Predicted proteins were annotated by blastx searches against DNA sequences and blastp searches against predicted protein sequences for each contig using the standalone NCBI BLAST 2.2.28+ program [34]. The E-value cut off option was set to 0.005. All data were integrated into a set of relational databases by the FileMaker Pro software.

### 2.3 Homology modeling

SWISS-MODEL (http://swissmodel.expasy.org/) [35,36] was used in full-automatic mode to model the cysteine-rich core domain of VanabinX based on the solution structure of Vanabin2 (PDB ID: 1VFI) [28]. The Alphafold2 program was used to predict multimer formation between Vanabin2 and VanabinX [37]. We used the PyMOL Molecular Graphics System v1.5.0.4 (https://pymol.org/2/; Schrödinger, Inc., New York, NY, USA) to generate a pictorial representation of the structure. The SWISS PDB viewer software (http://www.expasy.org/spdbv/) was used for surface electrical potential mapping [35].

### 2.4 Semi-quantitative RT-PCR

Reverse transcription was performed using ReverTra Ace reverse transcriptase (Toyobo Co., Ltd., Japan) and the dT15 primer. Each PCR mixture contained reverse-transcribed DNA from 2 ng total RNA, forward and reverse primers (10 pmol each), dNTPs (2 nmol each), 1 × PCR buffer, and 0.5 U HybriPol DNA Polymerase (Meridian Bioscience, Inc., USA) in a total reaction volume of 10 μL. Primers for PCR are listed in Supplementary Table 3. After incubation at 94 °C for 2 min, 27–36 cycles of PCR were performed (30 s at 94 °C, 30 s at 52 °C, and 60 s at 72 °C). Amplified fragments were examined by 1.5% agarose gel electrophoresis and ethidium bromide staining. PCR cycle numbers were experimentally determined for each gene to give a significant amount of product without reaching saturation.

### 2.5 Preparation of recombinant protein and purification of VanabinX

To construct plasmids for the expression of fusion protein, a cDNA fragment of VanabinX spanning the coding region was amplified by PCR using specific primer sets with artificial restriction sites (Supplementary Table 3). The amplified fragments were digested with *Eco*RI and *Sal*I, then ligated into the corresponding site of pMal-p2x (New England BioLabs, Inc., USA). The coding regions were ligated to the coding region for maltose-binding protein (MBP) to produce a fusion protein. The plasmid was introduced into the *E*. *coli* BL21 host strain. An overnight culture of non-induced cells bearing expressing plasmids was diluted 1:10 in LB medium containing 50 μg mL^−1^ ampicillin and 1 mM IPTG (Fujifilm Waco Pure Chemical Corporation, Japan), and then cultured at 37 °C for 6 h. The bacterial cells were collected by centrifugation (2500×*g*, 10 min, 4 °C) and sonicated in a lysis buffer (10 mM sodium phosphate [pH 7.0], 30 mM NaCl, 0.25% Tween 20, 10 mM mercaptoethanol, 10 mM EDTA). The fusion protein was purified from this lysate on an amylose resin column according to the manufacturer's protocol (New England BioLabs). The purity of the fusion proteins was confirmed via SDS-PAGE. Protein concentrations were measured with a protein assay kit (Nakalai Tesque, Inc., Japan) using bovine serum albumin (Pierce Manufacturing Inc., USA) as a standard.

### 2.6 V-binding assay with immobilized metal ion affinity chromatography (IMAC)

IMAC analysis was done according to a previous study [38]. Vanadyl sulfate (VOSO_4_·nH_2_O, *n* = 3–4; 99.9%), along with MgCl_2_, CaCl_2_, MnCl_2_, FeCl_3_, CoSO_4_, CuCl_2_, and ZnCl_2_, were purchased from Fujifilm Wako Pure Chemical Corporation, Japan. Each metal was dissolved in deionized water (DW) at 1 mM and stored at room temperature.

Chelating Sepharose FF resin (250 μL; Thermo Fisher Scientific, USA) was washed with water and mixed with each metal solution in a 2 mL plastic tube. The resin was washed twice with distilled water and twice with binding buffer (100 mM NaCl, 20 mM Na phosphate, pH 7.4). Protein in binding buffer (∼100 μg mL^−1^) was mixed with the resin by rotation for 30 min at room temperature. Non-bound proteins were removed by centrifugation, then the resin was washed twice with binding buffer. Bound proteins and metal ions were eluted with 50 mM EDTA (pH 8.0). Non-bound and bound protein fractions were analyzed by SDS-PAGE and CBB staining.

### 2.7 Coupled NADPH oxidation assay

The NADPH oxidation assay was done according to a previous study [16]. Sodium orthovanadate (Na_3_VO_4_; 99.98%) was purchased from Sigma Aldrich Co. LLC, USA. Solid sodium orthovanadate was dissolved at 100 mM in DW, the pH was adjusted to 8.0, and the solution was heated at 95 °C until it became colorless. For each experiment, V(V) stock solution was diluted to 10 mM and mixed with 4 mM EDTA, pH 8.0. NADPH, reduced glutathione (GSH), and glutathione reductase (GR) were purchased from FujiFilm Wako Pure Chemical Corporation.

The assay buffer contained 200 μM NADPH, 0.25 U/mL GR, and 2 mM GSH. Each protein was added to a final concentration of 1 μM in each tube except for the negative control. V(V)-EDTA (10 mM Na_3_VO_4_, 4 mM EDTA, pH 8.0) solution was added to a final concentration of 2.5 mM V(V) and 1 mM EDTA, and the measurement of absorbance at 340 nm was immediately started. Reductase activity was monitored for 30 min and expressed as μM of NADPH oxidized using a molar extinction coefficient of 6200 M^−1^ cm^−1^ for NADPH. All experiments were performed at 20 °C.

## 3 Results

### 3.1 Transcriptome analysis of ascidian blood cells

To generate a catalog of genes expressed in the normal condition of blood cells of the V-rich ascidian *Ascidia sydneiensis samea*, we extracted blood cells from four individuals cultured in an aquarium with flowing natural seawater. RNA samples were extracted from blood cells isolated from each individual separately (Supplementary Table 1).

Two cDNA libraries were constructed from each mRNA sample with or without normalization. Shotgun DNA sequences were obtained from each cDNA library (Supplementary Table 2). The results of assembly, prediction of coding sequences, and homology searches are summarized in Supplementary Table 2. The biological sample Hoya1_S1 yielded the best number of predicted peptides and was chosen as the starting point for the protein database. From Hoya1_S1, a total of 155,128 predicted protein sequences met the following: sequences starting with methionine, with both blastx and blastp hits, and a difference of less than 15 amino acids or 10% sequence similarity compared to the best hit protein. We examined each predicted protein manually to determine whether the contig had only one open reading frame (ORF) or the longest ORF had blastp and blastx hits. We also examined the remaining predicted proteins that started with methionine or had a high count even if there were no blast hits. Finally, we finalized the protein database with 8532 predicted proteins (Supplementary Table 4).

We analyzed the effect of normalization by comparing the occurrence of each of the 8532 predicted proteins in each of normalized and unnormalized libraries. A total of 5647 proteins were found in both the normalized and unnormalized libraries, 1175 proteins were only in unnormalized libraries, and 1438 proteins were only in normalized libraries. The remaining 272 proteins were not found in Hoya1_S1 but were from the other three individuals. The highest counts were for fibrillin-2 (31,572 counts), ferritin heavy chain (26,510 counts), and translation initiation factor (12,880 counts). The average count of the 8532 proteins in Hoya1_S1 was 162, while the median was 64. Known V-related proteins are listed in Table 1.

**Table 1:** Vanadium-related proteins.

ID	name	Hoya1_S1^a^	Hoya3_S2^a^	Hoya4_S3^a^	Hoya6_S4^a^
8166	vanadium-binding protein Vanabin1		237		1097
254	vanadium-binding protein Vanabin2	473			
384	vanadium-binding protein Vanabin3	266		43	
135	vanadium-binding protein similar to Vanabin4	764			
8184	vanadium-binding protein similar to Vanabin4		29		676
1658	vanadium-binding protein VanabinP	59			107
7719	vanadium-binding protein VanabinX	1677	17	4046	290
8265	vanadium-binding protein VBP-129				265
66	vanadium-binding protein similar to 8265	1276			
300	glutathione s-transferase (GST)	409			
22	glycogen phosphorylase (GP)	1910			
8134	glycogen phosphorylase identical to 22			3156	
35	transketolase (TKL)	1802			
184	6-phosphogluconate dehydrogenase (6-PGDH)	534		2201	322
1488	natural resistance-associated macrophage protein (AsNramp)	53		28	109
8120	natural resistance-associated macrophage protein similar to 1488	110			
772	thioredoxin 1 (AsTrx1)	176			
2160	vanabin-interacting protein almost identical to VIP1	11		34	36
3134	vanabin-interacting protein similar to 2160	0			
5882	vanabin-interacting protein similar to 2160	0			
8522	vanabin-interacting protein, similar to 2160	37			
8523	vanabin-interacting protein similar to 2160	33			
8524	vanabin-interacting protein similar to 2160	82			
8525	vanabin-interacting protein similar to 2160	0			
8526	vanabin-interacting protein similar to 2160	0			
8527	vanabin-interacting protein similar to 2160	264			
8528	vanabin-interacting protein similar to 2160			0	
8529	vanabin-interacting protein similar to 2160			4	
8530	vanabin-interacting protein similar to 2160			18	
8531	vanabin-interacting protein similar to 2160			59	
8532	vanabin-interacting protein similar to 2160			18	

a Counts from RNA-seq analyses of each biological sample. Zero indicates counts less than 0.01.

V-binding proteins including vanabins (Vanabin1, 2, 3, 4 and P) [[10], [11], [12]] and glutathione s-transferase (GST) [13] were found. In addition to five vanabins that were previously reported, we identified another vanabin by homology and examined it in detail in this study. This novel vanabin, named VanabinX (ID 7719), was the most abundant among all vanabins examined in this study.

Enzymes belonging to the pentose phosphate pathway, such as glycogen phosphorylase [39], transketolase [40], and 6-phosphogluconate dehydrogenase [15], were counted in high numbers. A membrane V transporter (AsNramp) [17], redox protein (AsTrx) [26], and vanabin-interacting protein 1 (VIP1) [41] were also found, but in lower abundance. Several variants of VIP1 found in this analysis varied greatly in length and sequence, but their significance was unclear and they were not examined further in this study.

### 3.2 Sequence and structure of VanabinX

**Fig. 1:**
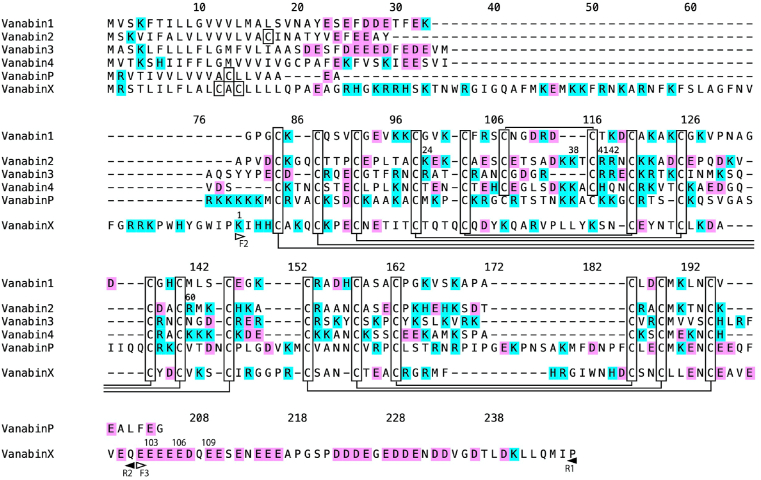
Alignment of amino acid sequences of vanabins identified from *Ascidia sydneiensis samea*. Conserved cysteines are boxed and putative disulfide bonds are indicated. VanabinX has a long C-terminal domain rich in acidic amino acids. Open and closed arrowheads indicate the positions of forward and reverse primers. Numbering of amino acid residues for the alignment starts from the initiation methionine; that of Vanabin2 is the same as in previous studies [28,38]; that of VanabinX starts from the lysine corresponding to the F2 primer position.

In contrast to previously reported vanabins, which possess 18 conserved cysteines in the core domain, VanabinX has only 16 cysteines (Fig. 1), as well as a long C-terminal stretch of acidic amino acids that is similar to those found in two vanabins from another ascidian, *Ciona intestinalis* type A (*Ciona robusta*) [23].

**Fig. 2:**
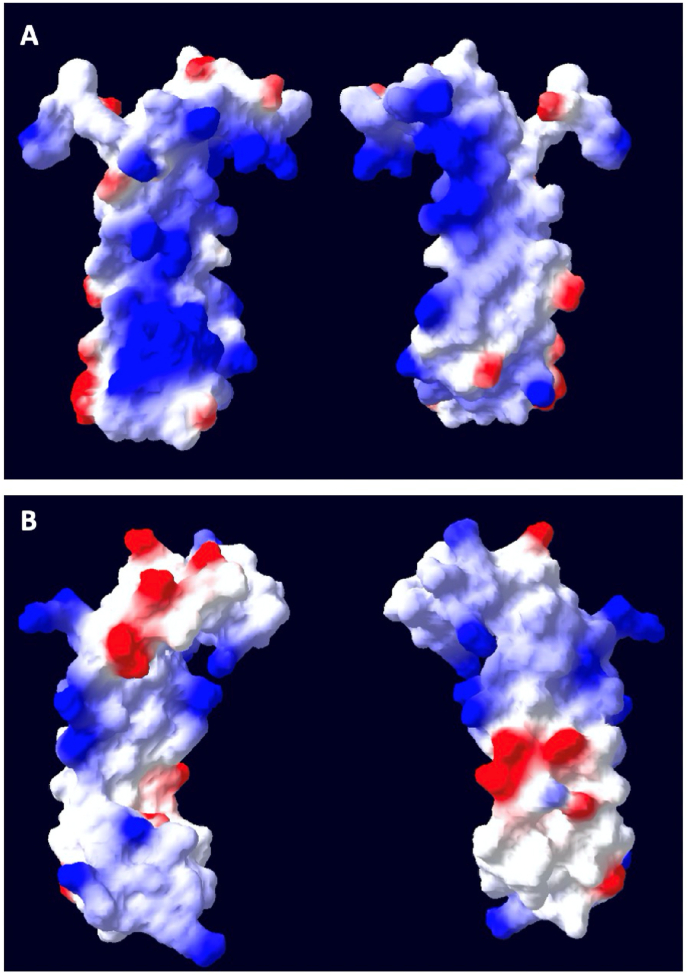
Comparison of structural models of the cysteine-rich core domains of (A) Vanabin2 and (B) VanabinX. Each panel depicts two opposite faces of the same molecule. The images were produced by SWISS PDB viewer with the following parameters: red −2.2, white 0.0, blue 4.0. Blue, positively charged; red, negatively charged. (A) Structure of Vanabin2 (PDB: 1vfi). (B) Structure of VanabinX (cysteine-rich core domain F2/R2) predicted by homology modeling based on the solution structure of Vanabin2. (For interpretation of the references to color in this figure legend, the reader is referred to the Web version of this article.)

We modeled the structure of VanabinX using the solution structure of Vanabin2 [28] as a template. Although the resulting structure is almost identical to that of Vanabin2, the electrostatic surface of VanabinX is more negative, reflecting the decreased number of lysine and arginine residues (Fig. 2). The relationships between the V(IV)-binding sites and sites of variation are discussed below.

### 3.3 Tissue localization of VanabinX

**Fig. 3:**
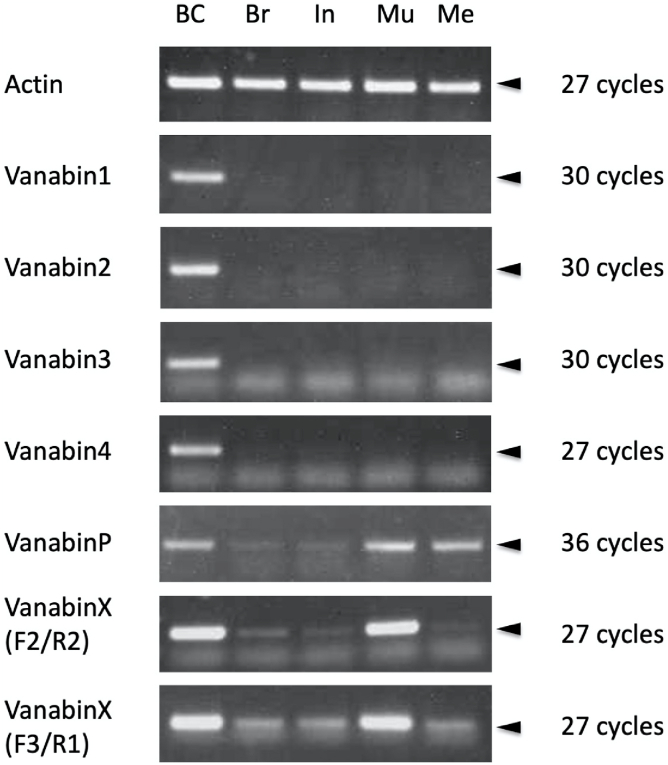
Tissue localization of Vanabins identified from *Ascidia sydneiensis samea*. Actin was included as a universal gene. BC: blood cells, Br: branchial sac, In: intestine, Mu: muscle, Me: mesenchyme. PCR products are indicated by arrowheads. PCR cycles are indicated for each gene on the right.

To examine the tissue localization of vanabins, we performed PCR for each gene using cDNAs derived from five major tissues of *Ascidia sydneiensis samea*. PCR primers for each gene are listed in Supplementary Tables 3 and a typical result of electrophoresis of PCR products is shown in Fig. 3. The universal control, cytoplasmic actin gene, was detected in all five tissues. Vanabin1, Vanabin2, Vanabin3, and Vanabin4 were only detected in blood cells. VanabinP and VanabinX showed a similar expression profile; both genes were highly expressed in blood cells and muscle but were also detected in the other three tissues. The expression of VanabinX was confirmed by two set of primers (F2/R2 and F3/R1).

### 3.4 Metal-binding assay for VanabinX

**Fig. 4:**
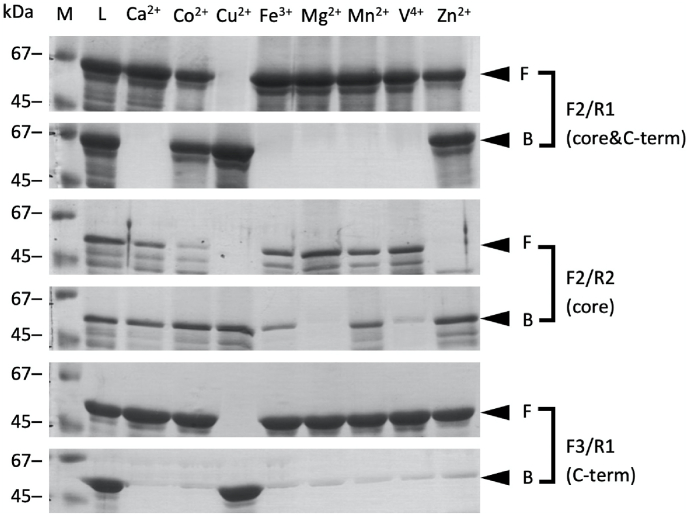
Metal selectivity and affinity of VanabinX fragments evaluated by IMAC. IMAC resins were charged with the metal ions indicated above. Proteins were applied to resins and non-binding fractions (F) were eluted with the equilibration buffer. Binding fractions (B) were subsequently eluted with elution buffer, and the proteins were analyzed by SDS-PAGE and staining with CBB. The names of primer sets are used to indicate the fusion proteins. (M) Molecular weight markers, (L) protein equivalents to those loaded to the column.

We performed IMAC to examine the metal-binding ability of VanabinX. Based on experimental evidence for Vanabin1, 2, and P [10,11], we presumed that the N-terminal domain is removed to make mature VanabinX with the cysteine-rich core domain and the C-terminal acidic domain and determined the position of the forward primer F2 (Fig. 1). Then, we prepared the cysteine-rich core domain and C-terminal acidic domain separately or in combination to assess the function of each domain. Namely, primers F2/R2 were used to obtain the cysteine-rich core domain, primers F3/R1 for the C-terminal acidic domain, and primers F2/R1 for their combination (Fig. 1) as recombinant proteins fused to maltose binding protein (MBP). The names of primer sets are used to indicate the fusion proteins. The fusion proteins were subjected to IMAC (Fig. 4).

The putative mature protein (F2/R1) bound strongly to Cu(II) and moderately to Co(II) and Zn(II). The cysteine-rich core domain (F2/R2) also bound to Co(II), Cu(II), and Zn(II). In addition, this domain (F2/R2) also bound to Ca(II), Fe(III), Mn(II), and V(IV). The C-terminal domain (F3/R1) weakly bound to all metal ions examined. These results suggest that the C-terminal domain (F3/R1) suppressed the binding of the core domain (F2/R2) to these metal ions and that putative mature VanabinX cannot bind to V(IV). Because the core domain (F2/R2) is susceptible to degradation, the C-terminal domain (F3/R1) might also have a protective function. It remains unclear whether VanabinX can bind directly to Cu(II) because MBP binds strongly to Cu(II) [42].

### 3.5 V(V) reduction by VanabinX coupled with NADPH oxidation

**Fig. 5:**
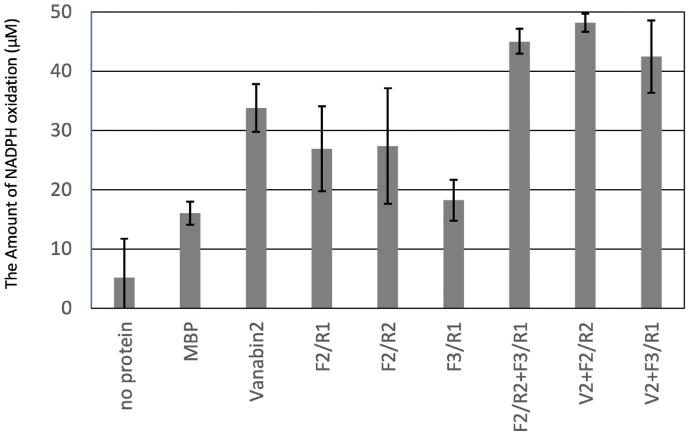
V(V) reduction as assessed by a NADPH coupled assay, measured as the NADPH oxidation in 30 min at 20 °C with 1 μM each protein or no protein control. Vanabin2 and VanabinX (F2/R1, F2/R2, and F3/R1) are fused to MBP. The assay buffer contained 200 μM NADPH, 0.25 U/mL GR, 2 mM GSH, 2.5 mM V(V), and 1 mM EDTA. Bars indicate mean ± S.D. (n = 6–13) for a summary of at least two technical repeats for at least two batches of protein preparation for each protein.

Vanabin2 reduces V(V) to V(IV) [16]. To determine whether VanabinX exhibits V(V)-reductase activity, we performed a coupled NADPH oxidation assay (Fig. 5). As MBP fusion proteins were also used in this assay, MBP was used as a control.

MBP showed significant V(V)-reductase activity, with NADPH oxidation (16.0 ± 2.0 μM V per micromolar protein within 30 min) compared to the no-protein control (P < 0.01, Student's two-tailed *t*-test). The V(V)-reductase activity of MBP-Vanabin2 and the two fragments for MBP-VanabinX (F2/R1 and F2/R2) was significantly greater than that of MBP (33.8 ± 4.0, 26.9 ± 7.1, and 27.4 ± 9.7 μM V per micromolar protein within 30 min, respectively; P < 0.02); however, the C-terminal fragment (F3/R1) did not show significant activity. When VanabinX (F2/R2) was mixed with the C-terminal domain (F3/R1), V(V)-reductase activity was significantly enhanced (165%; P < 0.001). Enhanced activity was also observed when Vanabin2 was mixed with F3/R1 (125%; P < 0.001). Because the activities of F2/R1 and F2/R2 were not significantly different, the V(V)-reductase activity of the cysteine-rich core domain (F2/R2) was likely not affected by the C-terminal domain (F3/R1) in *cis* mode. These results suggest that the C-terminal domain (F3/R1) enhances the V(V)-reductase activity in *trans* mode.

## 4 Discussion

### 4.1 Identification and expression of vanabins and V-related proteins

In this study, we performed transcriptome analysis of *A*. *sydneiensis samea* blood cells. We constructed a protein database containing 8532 predicted proteins (Supplementary Table 4). In addition to the five previously reported vanabins, we identified one vanabin through homology and examined it in detail in this study. This novel vanabin, named VanabinX, was the most abundant among all vanabins (Table 1). Previous studies may have failed to detect VanabinX despite its abundance due to differences in mRNA stability, the mRNA translation rate, and the protein degradation rate among vanabins; the lack of V(IV)-binding activity in VanabinX is another possible explanation.

Enzymes belonging to the pentose phosphate pathway, such as glycogen phosphorylase, transketolase, and 6-phosphogluconate dehydrogenase, were also highly abundant (Table 1), allowing us to identify these proteins in a random screening of monoclonal antibodies that react with blood cells [14,15]. RT-PCR showed that VanabinP and VanabinX had similar expression profiles (Fig. 3). In contrast to the blood cell–specific expression of other vanabins, VanabinP and VanabinX were highly expressed in blood cells and muscle tissue, and were also detected in three other tissue types. VanabinP is secreted into blood plasma and acts as a V(IV) carrier in the blood; its function in other tissue types remains unknown. Further study is required to determine the functions of vanabins in tissues other than blood cells.

### 4.2 Structure and function of VanabinX

The overall structure of the core domain of VanabinX (F2/R2) is similar to Vanabin2, but the surface charges are quite different (Fig. 2). Vanabin2 binds to V(IV) by amine nitrogens of amino acid residues such as lysines and arginines in the cysteine-rich core domain [43]. Two V(IV) binding sites have been identified by site-directed mutagenesis on Vanabin2 (K10, K24, K38, R41, and R42) [38]. VanabinX does not have any of the lysines or arginines in these sites. If we assume that ancestral vanabin has the V(IV) binding ability, the loss of these amino acids may account for the decrease in V(IV) binding ability in VanabinX.

**Fig. 6:**
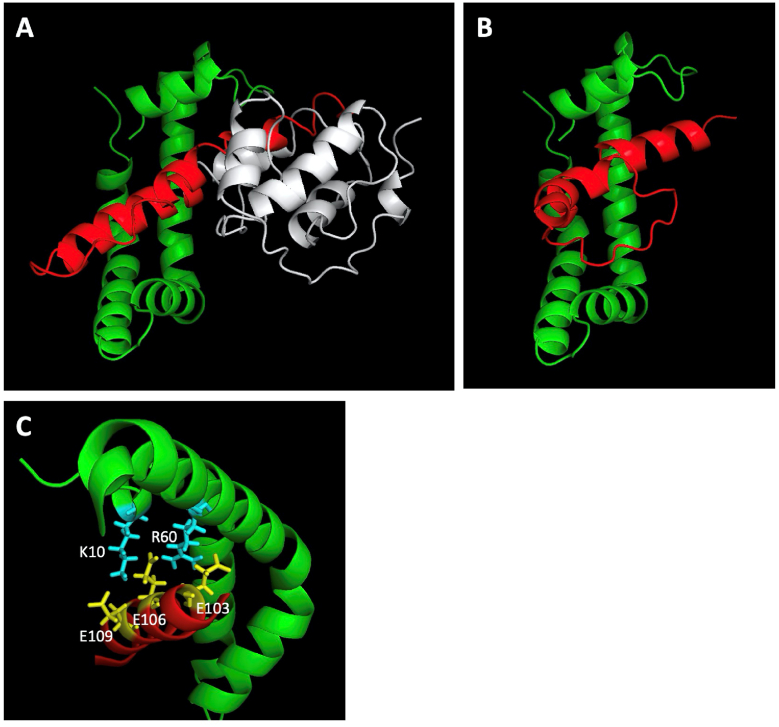
Multimeric structural models predicted by the Alphafold2 software. The images were produced by the PyMol software. (A, B) Vanabin2 modeled with VanabinX fragments F2/R1 (A) or F3/R1 (B). Green: Vanabin2, gray: VanabinX core domain (F2/R2), red: VanabinX C-terminal acidic domain (F3/R1). (C) Close up of multimers in (A) showing the V(IV)-coordination site 1 made from lysines (K10) and arginine (R60) in Vanabin2 and three glutamic acid residues (E103, E106, E109) in the VanabinX C-terminal domain. Cyan: lysine and arginine, yellow: glutamate. (For interpretation of the references to color in this figure legend, the reader is referred to the Web version of this article.)

The C-terminal acidic amino acid-rich domain (F3/R1) is unique to VanabinX and has an enhancing activity for the V(V)-reductase activity of Vanabin2 (Fig. 5). When this domain is combined with Vanabin2, it fits perfectly into the V(IV)-binding pocket of the bow-shaped conformation of Vanabin2 (Fig. 6). Because the lysine and arginine at V(IV)-binding site 1 (K10 and R60) are close to carboxyl residues of the three glutamic acid residues (E101, E103, and E106) of VanabinX, ionic interaction might affect the overall structure and result in the enhancement the V(V)-reductase activity.

### 4.3 Roles of VanabinX in V accumulation and reduction in blood cells

In our model, V(V) ions were readily reduced to V(IV) in the cytoplasm by vanabins, and V(IV) ions were stabilized by vanabins [20]. Because putative full-length VanabinX (F2/R1) cannot bind to V(IV), only VanabinX enhanced the V(V) reductase activity of other vanabins. Because putative full-length VanabinX (F2/R1) was required to reduce V (Fig. 5), reduced V(IV) must be captured by other vanabins that bind to V(IV). Vanabin-interacting proteins (VIPs) [41] may assist such interactions; these processes require further study to clarify the interacting network of vanabins and VIPs involved in V reduction in blood cells.

The rates of V reduction and accumulation *in vivo* remain unknown, and the extent to which VanabinX enhances V accumulation *in vivo* is also unclear. Further studies involving the genetic modification of vanabins are necessary to clarify the contribution of each vanabin to this process.

## 5 Conclusions

We performed a transcriptome analysis of blood cells of the V-rich ascidian *Ascidia sydneiensis samea*. Among the 8532 predicted proteins, we found a novel vanabin and named it VanabinX. VanabinX has only 16 cysteines in the core region and lacks lysines that could make V(IV)-binding sites. VanabinX has a unique feature of a long C-terminal stretch of acidic amino acids. Biochemical assays suggested that VanabinX does not bind to V and that its C-terminal acidic domain enhances the V(V)-reductase activity of vanabins in *trans* mode. Thus, VanabinX serves as a V(V)-reductase enhancer in V accumulation in blood cells.

## Declaration of competing interest

The authors declare that they have no known competing financial interests or personal relationships that could influence the work reported in this paper.

## References

[1] Michibata H., Ueki T. (2012). Vanadium - Biochemical and Molecular Biological Approaches.

[2] Michibata H., Yoshinaga M., Yoshihara M., Kawakami N., Yamaguchi N., Ueki T. (2007). Pubs.Acs.org.

[3] Michibata H., Yamaguchi N., Uyama T., Ueki T. (2003). Molecular biological approaches to the accumulation and reduction of vanadium by ascidians. Coord. Chem. Rev..

[4] Michibata H., Iwata Y., Hirata J. (1991). Isolation of highly acidic and vanadium-containing blood cells from among several types of blood cell from Ascidiidae species by density-gradient centrifugation. J. Exp. Zool..

[5] Michibata H., Terada T., Anada N., Yamakawa K., Numakunai T. (1986). The accumulation and distribution of vanadium, iron, and manganese in some solitary ascidians. Biol. Bull..

[6] Hirata J., Michibata H. (1991). Valency of vanadium in the vanadocytes of *Ascidia gemmata* separated by density-gradient centrifugation. J. Exp. Zool..

[7] Ueki T., Takemoto K., Fayard B., Salomé M., Yamamoto A., Kihara H., Susini J., Scippa S., Uyama T., Michibata H. (2002). Scanning x-ray microscopy of living and freeze-dried blood cells in two vanadium-rich ascidian species, *Phallusia mammillata* and *Ascidia sydneiensis samea*. Zool. Sci..

[8] Michibata H., Hirata J., Uesaka M. (1987). Separation of vanadocytes: determination and characterization of vanadium ion in the separated blood cells of the ascidian, *Ascidia ahodori*. J. Exp. Zool..

[9] Kanda T., Nose Y., Wuchiyama J., Uyama T., Moriyama Y., Michibata H. (1997). Identification of a vanadium-associated protein from the vanadium-rich ascidian, *Ascidia sydneiensis samea*. Zool. Sci..

[10] Ueki T., Adachi T., Kawano S., Aoshima M., Yamaguchi N., Kanamori K., Michibata H. (2003). Vanadium-binding proteins (vanabins) from a vanadium-rich ascidian *Ascidia sydneiensis samea*. Biochim. Biophys. Acta.

[11] Yoshihara M., Ueki T., Watanabe T., Yamaguchi N., Kamino K., Michibata H. (2005). VanabinP, a novel vanadium-binding protein in the blood plasma of an ascidian, *Ascidia sydneiensis samea*. Biochim. Biophys. Acta.

[12] Yamaguchi N., Kamino K., Ueki T., Michibata H. (2004). Expressed sequence tag analysis of vanadocytes in a vanadium-rich ascidian, *Ascidia sydneiensis samea*. Mar. Biotechnol..

[13] Yoshinaga M., Ueki T., Yamaguchi N., Kamino K., Michibata H. (2006). Glutathione transferases with vanadium-binding activity isolated from the vanadium-rich ascidian *Ascidia sydneiensis samea*. Biochim. Biophys. Acta.

[14] Uyama T., Yamamoto K., Kanamori K., Michibata H. (1998). Glucose-6-phosphate dehydrogenase in the pentose phosphate pathway is localized in vanadocytes of the vanadium-rich ascidian, *Ascidia sydneiensis samea*. Zool. Sci..

[15] Uyama T., Kinoshita T., Takahashi H., Satoh N., Kanamori K., Michibata H. (1998). 6-Phosphogluconate dehydrogenase is a 45-kDa antigen recognized by S4D5, a monoclonal antibody specific to vanadocytes in the vanadium-rich ascidian *Ascidia sydneiensis samea*. J. Biochem..

[16] Kawakami N., Ueki T., Amata Y., Kanamori K., Matsuo K., Gekko K., Michibata H. (2009). A novel vanadium reductase, Vanabin2, forms a possible cascade involved in electron transfer. Biochim. Biophys. Acta.

[17] Ueki T., Furuno N., Michibata H. (2011). A novel vanadium transporter of the Nramp family expressed at the vacuole of vanadium-accumulating cells of the ascidian *Ascidia sydneiensis samea*. Biochim. Biophys. Acta.

[18] Ueki T., Furuno N., Xu Q., Nitta Y., Kanamori K., Michibata H. (2009). Identification and biochemical analysis of a homolog of a sulfate transporter from a vanadium-rich ascidian *Ascidia sydneiensis samea*. Biochim. Biophys. Acta.

[19] Ueki T., Yamaguchi N., Michibata H. (2003). Chloride channel in vanadocytes of a vanadium-rich ascidian *Ascidia sydneiensis samea*. Comp. Biochem. Physiol..

[20] Ueki T., Yamaguchi N., Romaidi, Isago Y., Tanahashi H. (2015). Vanadium accumulation in ascidians: a system overview. Coord. Chem. Rev..

[21] Ueki T., Michibata H. (2011). Molecular mechanism of the transport and reduction pathway of vanadium in ascidians. Coord. Chem. Rev..

[22] Dehal P. (2002). The draft genome of *Ciona intestinalis*: insights into chordate and vertebrate origins. Science.

[23] Trivedi S., Ueki T., Yamaguchi N., Michibata H. (2003). Novel vanadium-binding proteins (vanabins) identified in cDNA libraries and the genome of the ascidian *Ciona intestinalis*. Biochim. Biophys. Acta.

[24] Yamaguchi N., Togi A., Ueki T., Uyama T., Michibata H. (2002). Expressed sequence tag analysis of blood cells in the vanadium-rich ascidian, *Ascidia sydneiensis samea*–A survey of genes for metal accumulation. Zool. Sci..

[25] Samino S., Michibata H., Ueki T. (2012). Identification of a novel Vanadium-binding protein by EST analysis on the most vanadium-rich ascidian, *Ascidia gemmata*. Mar. Biotechnol..

[26] Ueki T., Uwagaki M., Yamamoto S., Michibata H. (2014). Participation of thioredoxin in the V(V)-reduction reaction by Vanabin2. Biochim. Biophys. Acta.

[27] Kitayama H., Yamamoto S., Michibata H., Ueki T. (2013). Metal ion selectivity of the vanadium (V)-reductase Vanabin2. Dalton Trans..

[28] Hamada T., Asanuma M., Ueki T., Hayashi F., Kobayashi N., Yokoyama S., Michibata H., Hirota H. (2005). Solution structure of Vanabin2, a vanadium(IV)-binding protein from the vanadium-rich ascidian *Ascidia sydneiensis samea*. J. Am. Chem. Soc..

[29] Smale G., Sasse J. (1992). RNA isolation from cartilage using density gradient centrifugation in cesium trifluoroacetate: an RNA preparation technique effective in the presence of high proteoglycan content. Anal. Biochem..

[30] Chomczynski P., Sacchi N. (1987). Single-step method of RNA isolation by acid guanidinium thiocyanate-phenol-chloroform extraction. Anal. Biochem..

[31] Matvienko M., Kozik A., Froenicke L., Lavelle D., Martineau B., Perroud B., Michelmore R. (2013). Consequences of normalizing transcriptomic and genomic libraries of plant genomes using a duplex-specific nuclease and tetramethylammonium chloride. PLoS ONE.

[32] Grabherr M.G., Haas B.J., Yassour M., Levin J.Z., Thompson D.A., Amit I., Adiconis X., Fan L., Raychowdhury R., Zeng Q., Chen Z., Mauceli E., Hacohen N., Gnirke A., Rhind N., Di Palma F., Birren B.W., Nusbaum C., Lindblad-Toh K., Friedman N. (2011). Trinity: reconstructing a full-length transcriptome without a genome from RNA-Seq data. Nat. Biotechnol..

[33] Li B., Dewey C.N. (2011). RSEM: accurate transcript quantification from RNA-Seq data with or without a reference genome. BMC Bioinformatics.

[34] Altschull S.F., Gishl W., Miller W., Myers E.W., Lipman D.J. (1990). Basic local alignment search tool. J. Mol. Biol..

[35] Guex N., Peitsch M.C. (1997). SWISS-MODEL and the Swiss-Pdb Viewer: an environment for comparative protein modeling. Electrophoresis.

[36] Shwede T., Kopp J., Guex N., Peitsch M.C. (2003). SWISS-MODEL: an automated protein homology-modeling server. Nucl. Acids Res..

[37] Jumper J., Evans R., Pritzel A., Green T., Figurnov M., Ronneberger O., Tunyasuvunakool K., Bates R., Žídek A., Potapenko A., Bridgland A., Meyer C., Kohl S.A.A., Ballard A.J., Cowie A., Romera-Paredes B., Nikolov S., Jain R., Adler J., Back T. (2021). Highly accurate protein structure prediction with AlphaFold. Nature.

[38] Ueki T., Kawakami N., Toshishige M., Matsuo K., Gekko K., Michibata H. (2009). Characterization of vanadium-binding sites of the vanadium-binding protein Vanabin2 by site-directed mutagenesis. Biochim. Biophys. Acta..

[39] Uyama T., Ueki T., Suhama Y., Kanamori K., Michibata H. (1998). A 100-kDa antigen recognized by a newly prepared monoclonal antibody specific to the vanadocytes of the vanadium-rich ascidian, *Ascidia sydneiensis samea*, is glycogen phosphorylase. Zool. Sci..

[40] Ueki T., Uyama T., Yamamoto K., Kanamori K., Michibata H. (2000). Exclusive expression of transketolase in the vanadocytes of the vanadium-rich ascidian, *Ascidia sydneiensis samea*. Biochim. Biophys. Acta.

[41] Ueki T., Shintaku K., Yonekawa Y., Takatsu N., Yamada H., Hamada T., Hirota H., Michibata H. (2007). Identification of Vanabin-interacting protein 1 (VIP1) from blood cells of the vanadium-rich ascidian *Ascidia sydneiensis samea*. Biochim. Biophys. Acta.

[42] Lee K.-M., Ma K.-W., Shaw P.-C., Wong K.-B. (2006). A high-yield one-step purification method using copper-chelating chromatography for recombinant proteins fused with maltose-binding protein. Anal Biochem.

[43] Fukui K., Ueki T., Ohya H., Michibata H. (2003). Vanadium-binding protein in a vanadium-rich ascidian *Ascidia sydneiensis samea*: CW and pulsed EPR studies. J. Am. Chem. Soc..

