# Supplementary material for: The acidic amino acid-rich C-terminal domain of VanabinX enhances reductase activity, attaining 1.3- to 1.7-fold vanadium reduction
Source: Biochem Biophys Rep. 2022 Sep 16;32:101349. doi: 10.1016/j.bbrep.2022.101349 (PMC9486056; doi:10.1016/j.bbrep.2022.101349)
Supplement: Multimedia component 1 [file mmc1.docx]

Supplementary Table 1. RNAs extracted from blood cells from four individuals of *Ascidia sydneiensis samea*.

| serial ID | biological sample ID | body color | volume of blood  (mL) | amount of extracted RNA  (µg) |
| --- | --- | --- | --- | --- |
| #1 | Hoya1_S1 | white | 1.5 | 1.91 |
| #2 | Hoya3_S2 | brown | 3.5 | 1.30 |
| #4 | Hoya4_S3 | red | 5.5 | 3.57 |
| #5 | Hoya6_S4 | red orange | 5.0 | 3.43 |

Supplementary Table 2. Detail of DNA sequencing, assembling, translation and homology searches.

| biological sample ID | Hoya1_S1 | | Hoya3_S2 | | Hoya4_S3 | | Hoya6_S4 | |
| --- | --- | --- | --- | --- | --- | --- | --- | --- |
| normalization | no | yes | no | yes | no | yes | no | yes |
| RNA-seq dataset ID | Hoya1_S1 | Hoya1_Nor_S1 | Hoya3_S2 | Hoya3_Nor_S2 | Hoya4_S3 | Hoya4_Nor_S3 | Hoya6_S4 | Hoya6_Nor_S4 |
| count (pairs) | 8,982,326 | 7,870,406 | 8,805,747 | 8,331,540 | 8,586,367 | 6,825,706 | 8,768,651 | 7,527,682 |
| Trinity count (isoforms) | 73,941 | 8,1187 | 15,359 | 32,025 | 58,043 | 63,524 | 40,281 | 69,212 |
| N25 | 3,388 | 3,482 | 870 | 960 | 3,970 | 3,848 | 2,312 | 2,981 |
| N50 | 2,011 | 2,069 | 462 | 490 | 2,374 | 2,313 | 1,148 | 1,568 |
| N75 | 953 bp | 966 | 301 | 302 | 1,129 | 1,066 | 498 | 614 |
| GC % | 41.9 | 41.78 | 47.17 | 46.35 | 43.61 | 43.58 | 44.63 | 43.95 |
| with blastx hits | 34,399 | 37,334 | 7,936 | 15,622 | 28,668 | 31,472 | 20,929 | 32,778 |
|  | 47% | 46% | 52% | 49% | 49% | 50% | 52% | 47% |
| transcoder peptide count | 48,113 | 56,146 | 7,673 | 13118 | 40,500 | 43,541 | 22,434 | 37,479 |
| with blastp hits | 32,816 | 37,548 | 6,032 | 10,364 | 31,061 | 37,572 | 18,717 | 31,092 |
| with identical blastx and blastp hits | 31,913 | 36,500 | 5,809 | 9,656 | 28,632 | 31,083 | 17,192 | 27,390 |

Supplementary Table 3. Primer list.

| gene | primer ID | primer sequence* | expected fragment size | transcriptome ID | reference |
| --- | --- | --- | --- | --- | --- |
| Actin | F  R | GACCTATGCTGCTCTTGA  CAGGATGGATCCTCCAAT | 391 bp | 00003 | {Yoshihara:2005eu} |
| Vanabin1 | 12F2  12R | GGAATTCGGCCCAGGCTGCAAA  CGTCGACCTACACACAATTCAA | 278 bp | 08166 | {Ueki:2003wz} |
| Vanabin2 | 15F2  15R | GGAATTCGCTCCGGTGGATTGC  CGTCGACCTACTTGCAGTTTGTC | 290 bp | 00254 | {Ueki:2003wz} |
| Vanabin3 | 3-N  3-C | GAATTCCTACCCTGAATGTGATTG  CTCGAGTCAAAATCGAAGATGAC | 274 bp |  | {Yamaguchi:2004dq} |
| Vanabin4 | F1  R1 | TTTTCCTTGGCATGGTTGTGGT  CTTCGCAACTTGATTTGCAGTTGG | 297 bp |  | {Yamaguchi:2004dq} |
| VanabinP | F1  R3 | GAATTCCGAAAGAAAAAGAAG  GTCGACTCAACCTTCAAACAA | 395 bp | 01658 | {Yoshihara:2005eu} |
| VanabinX | F2  R1 | GGAATTCAAGATACATCACTGCCGTAAGC  GTCGACTCAGGGAATCATTTGAAGGAG | 454 bp | 07719 | this study |
| VanabinX | F2  R2 | same as above  CGTCGACTCAAACCGCTTCGCAGTTC | 308 bp | 07719 | this study |
| VanabinX | F3  R1 | GGAATTCGAAGTGGAGCAGAAGAG  same as above | 163 bp | 07719 | this study |

*Underlined residues correspond to artificial restriction sites.
